# Establishing standards of care for forensic mental health: an international Delphi consensus-building study

**DOI:** 10.3389/fpsyt.2026.1802511

**Published:** 2026-04-16

**Authors:** Marichelle Leclair, Arianne Imbeault, Brian McKenna, Tonia Nicholls, Anne Crocker, Lindsay Thomson

**Affiliations:** 1 Department of Psychoeducation and Psychology, Université du Québec en Outaouais, Gatineau, QC, Canada; 2 Institut national de psychiatrie légale Philippe-Pinel, Montréal, QC, Canada; 3 Department of Psychology, Université de Montréal, Montréal, QC, Canada; 4 Auckland Regional Forensic Psychiatry Services, Auckland, New Zealand; 5 School of Clinical Sciences, Auckland University of Technology, Auckland, New Zealand; 6 Centre for Forensic Behavioural Science, Swinburne University of Technology, Melbourne, VIC, Australia; 7 Department of Psychiatry, University of British Columbia, Vancouver, BC, Canada; 8 British Columbia Mental Health & Substance Use Services, Coquitlam, BC, Canada; 9 Department of Psychiatry and Addictology, Université de Montréal, Montréal, QC, Canada; 10 The State Hospital, Carstairs, United Kingdom; 11 University of Edinburgh, Edinburgh, United Kingdom

**Keywords:** consensus statement, Delphi, forensic mental health services, service organization, standards of care

## Abstract

**Objectives:**

The present study aimed to establish a consensus on a definition of forensic mental health systems and services, and to identify principles and components of forensic mental health systems.

**Methods:**

A Delphi consensus-building process was employed among 23 experts in forensic mental health, defined by lived experience of forensic mental health services, professional, clinical or management practice in forensic settings, or academic research in the field. Items were rated on a 9-point Likert scale, with consensus defined as ≥75% of panelists rating an item between 7 and 9. Across three Delphi rounds, items were revised, merged, or added based on participant feedback. Data were collected anonymously using LimeSurvey, with reminders sent to maximize participation, followed by a structured consensus meeting to resolve remaining areas of disagreement.

**Results:**

The final consensus statement comprises three components: (1) a definition of forensic mental health services; (2) a general statement including 12 guiding principles; and (3) 43 core components organized across 10 thematic domains addressing models of care, pathways and processes, programs and activities, physical health, service user and peer involvement, evaluation and improvement, service integration, safe environments, restrictive practices, and other system-level considerations. While all items achieved consensus at the consensus meeting, areas of sustained discussion related to the integration of cultural expertise, the inclusion of a lived experience workforce, and the distinction between descriptive and aspirational elements of forensic mental health services.

**Conclusions:**

This international consensus statement provides a structured framework for understanding forensic mental health systems. By articulating shared principles and core components while allowing flexibility across jurisdictions, the framework offers a foundation to support service development and evaluation across diverse jurisdictions.

## Introduction

Across jurisdictions, forensic mental health systems carry a dual mandate: supporting recovery of people under their care, while ensuring the safety of service users, health workers, and the public (1–3). Over the past two decades, forensic mental health systems have experienced sustained pressure associated to rising demand (2, 4) and complexity of service user profiles (5). The fragilization of forensic mental health systems has been accelerated by the COVID-19 pandemic, with profound, longlasting structural transformations in service provision and implementation (6–9) challenging their capacity to offer safe, appropriate and timely services (10, 11).

Despite these shared pressures, the organization of forensic mental health systems varies substantially across jurisdictions as a product of historical, legislative, and ongoing sociopolitical, cultural, and economic forces (1). Jurisdictions can differ, among others, in how forensic and general mental health services are integrated, how governance and accountability are structured, and how care is distributed across levels of security. Although scholars have speculated about the relative advantages and limitations of different models for configuring forensic mental health systems (1, 12–16), this area has rarely been subjected to empirical scrutiny (17).

The absence of empirically grounded guidance presents a significant challenge for jurisdictions seeking to develop, reform, evaluate, or compare their forensic mental health services. Indeed, large-scale, international studies remain difficult to conduct given the complexity of forensic systems, differences in legal frameworks, and variability in available data (1). At a more fundamental level, such studies at this time are limited by the lack of a shared understanding of what constitutes a forensic mental health system and which components should be considered core. In this context, an international Delphi consensus-building approach provides a pragmatic first step, creating a common understanding upon which future large-scale comparative research can be built. The present study thus aimed to establish a consensus on a definition of forensic mental health systems and services, and to identify principles and components of forensic mental health systems.

## Methods

### Design

We used a three-round international Delphi consensus-building process, a widely used method in health services research to develop common definitions, guidelines, or standards of care (18). The study was approved by the ethics committees of the Université du Québec en Outaouais, Canada as well as the National Health Service Medical Research Ethics Committee, United Kingdom. We followed the guidelines outlined in the Conducting and REporting of DElphi Studies (CREDES) (19).

### Panelists

Panelists were recruited using a purposeful sampling approach across multiple countries, including Argentina, Australia, Brazil, Canada, China, Denmark, Finland, Germany, Ireland, Italy, the Netherlands, New Zealand, Nigeria, Scotland, Sweden, the United Kingdom, and the United States. To be invited, potential panelists were required to be recognized as experts in forensic mental health services. Expertise was defined as having lived experience of forensic mental health services, working as a professional, clinician, or manager in forensic mental health settings (e.g., psychiatrist, psychologist, nurse), or conducting research in the field as an academic (e.g., professor). Other inclusion criteria included being 18 years old or above, and able to complete a questionnaire in English. Exclusion criteria included being unable to consent to research or lacking internet access.

A list of participants was generated by selecting members from the International Association of Forensic Mental Health Services (IAFMHS), an international non-profit organization that brings together researchers, clinicians, and other professionals in forensic mental health. This list was compiled to ensure balanced representation across professional roles, types of experience, and geographic regions. The preliminary list was subsequently reviewed by the full research team to identify any potential gaps in representation or areas of missing expertise. This process allowed the greater inclusion of experts with lived experience, who were recruited through the organizations and service user committees where the co-investigators were affiliated. These experts were invited either via large-scale emails or through personal, targeted invitations from the research team.

A pre-recruitment email was sent to 51 potential panelists across 11 countries, of whom 30 consented to participate (59%). This rate is comparable to other international Delphi studies (20). All rounds of Delphi questionnaires were sent to panelists who responded positively to the pre-recruitment invitation. A total of 23 unique panelists took part, with 19 participating in Round 1, 15 in Round 2, and 12 in Round 3. **Table 1** describes the panelists at all steps of the Delphi process.

**Table 1:** Description of panelists.

Round	N	Source of expertise	Management role in forensic	Countries
Recruitment	30	Lived experience expertise, n = 7 Clinical expertise, n = 3 Research expertise, n = 5 Combined clinical and research expertise, n = 15	Yes: n = 18 No: n = 12	Australia, n = 2 Canada, n = 11 Denmark, n = 2 Germany, n = 1 Netherlands, n = 1 New Zealand, n = 6 Nigeria, n = 1 Scotland, n = 1 Sweden, n = 2 England and Wales, n = 2 United States of America, n = 1
Round 1	19	Lived experience expertise, n = 5 Clinical expertise, n = 2 Research expertise, n = 4 Combined clinical and research expertise, n = 8	Yes: n = 12 No: n = 7	Australia, n = 2 Canada, n = 5 Denmark, n = 1 Germany, n = 1 Netherlands, n = 1 New Zealand, n = 4 Nigeria, n = 1 Scotland, n = 1 Sweden, n = 1 England and Wales, n = 2
Round 2	15	Lived experience expertise, n = 3 Clinical expertise, n = 3 Research expertise, n = 1 Combined clinical and research expertise, n = 8	Yes: n = 10 No: n = 5	Australia, n = 2 Canada, n = 2 Denmark, n = 2 Germany, n = 1 New Zealand, n = 5 Scotland, n = 1 Sweden, n = 2
Round 3	13	Lived experience expertise, n = 4 Clinical expertise, n = 1 Research expertise, n = 1 Combined clinical and research expertise, n = 7	Yes: n = 8 No: n = 5	Australia, n = 1 Canada, n = 5 Germany, n = 1 New Zealand, n = 3 Scotland, n = 1 Sweden, n = 2
Consensus meeting	7	Lived experience expertise, n = 1 Clinical expertise, n = 1 Research expertise, n = 3 Combined clinical and research expertise, n = 2	Yes: n = 6 No: n = 1	Canada, n = 4 Denmark, n = 1 New Zealand, n = 1 Scotland, n = 1

### Procedure

#### Questionnaire development

For the initial definition of forensic mental health services, we used a version that had been developed by the special interest group in Service Delivery and Development of the International Association of Forensic Mental Health Services (IAFMHS), a group for administrators, directors, and service managers in forensic mental health. For core components, an initial draft was developed by the first two authors. Consistent with other Delphi in health services research (e.g., 21), we used two sources of information.

First, we conducted a structured narrative review of the international scientific literature on forensic mental health service organization. We focused on peer-reviewed theoretical and qualitative work examining system-level models of care, service architecture, governance structures, and mechanisms associated with recovery and public safety (14, 22–28). Sources were identified through targeted searches in PsycINFO, PubMed, and Google Scholar, as well as through foundational reviews of forensic mental health service organization (1, 29). The aim was not to conduct a systematic review, but to identify recurring conceptual and structural components of forensic mental health systems that could be translated into Delphi items.

In addition, secondary use was made of qualitative data drawn from semi-structured interviews conducted at The State Hospital, a high-security forensic psychiatric hospital in Scotland. This institution was selected because it had recently been the subject of a formal service evaluation and was actively engaged in reflecting on how its model of care functioned in practice (30). One focus group and 14 individual semi-structured interviews were conducted in English with service users (n = 5), staff members (n = 10), managers (n = 3), and an advocacy representative (n = 1).

Service users were eligible if they had received services within the forensic mental health system for at least six months, were 18 years of age or older, and were able to speak English. Recruitment of service users took place in collaboration with administrative and clinical leaders, with care teams initially identifying and approaching potential panelists; informed consent was subsequently obtained directly by the interviewer. Staff panelists were identified in collaboration with clinical directors to ensure representation across a range of professional roles, while administrators were recruited through targeted email invitations and supplemented using a snowball strategy until sufficient breadth of perspectives was achieved.

Interview guides explored panelists’ perspectives on what aspects of the service supported recovery and well-being, how specific practices and structures contributed to observed outcomes (e.g., recovery), perceived challenges facing the service, and areas where changes could improve effectiveness. All interviews were audio-recorded, transcribed verbatim, and analyzed using thematic analysis (31), with specific attention to identifying patterns related to contexts and mechanisms that were associated with perceived outcomes.

Finally, the other co-authors piloted and refined a test version of the Delphi using their in-depth knowledge of forensic systems in Scotland (Thomson), New Zealand (McKenna, Leclair), and Canada (British Columbia: Nicholls; Québec: Crocker, Imbeault, Leclair).

Drawing from the literature and the interviews, the first two authors constructed 48 items across 10 sections. After piloting and revising the initial set of items with the whole research team, the list was revised to 60 items across 12 thematic areas. The thematic areas were: (1) Definition, (2) Models of Care, (3) Underpinning Values, (4) Pathways and Processes, (5) Programs, Treatments, and Activities, (6) Physical Health, (7) Involvement of Service Users, Family, and Peers, (8) Evaluation and Improvement, (9) Collaboration between Services, (10) Safe Environment, (11) Restrictive Measures, and (12) Other. The initial Delphi questionnaire is available in the online supplement.

#### Rounds

**Figure 1:**
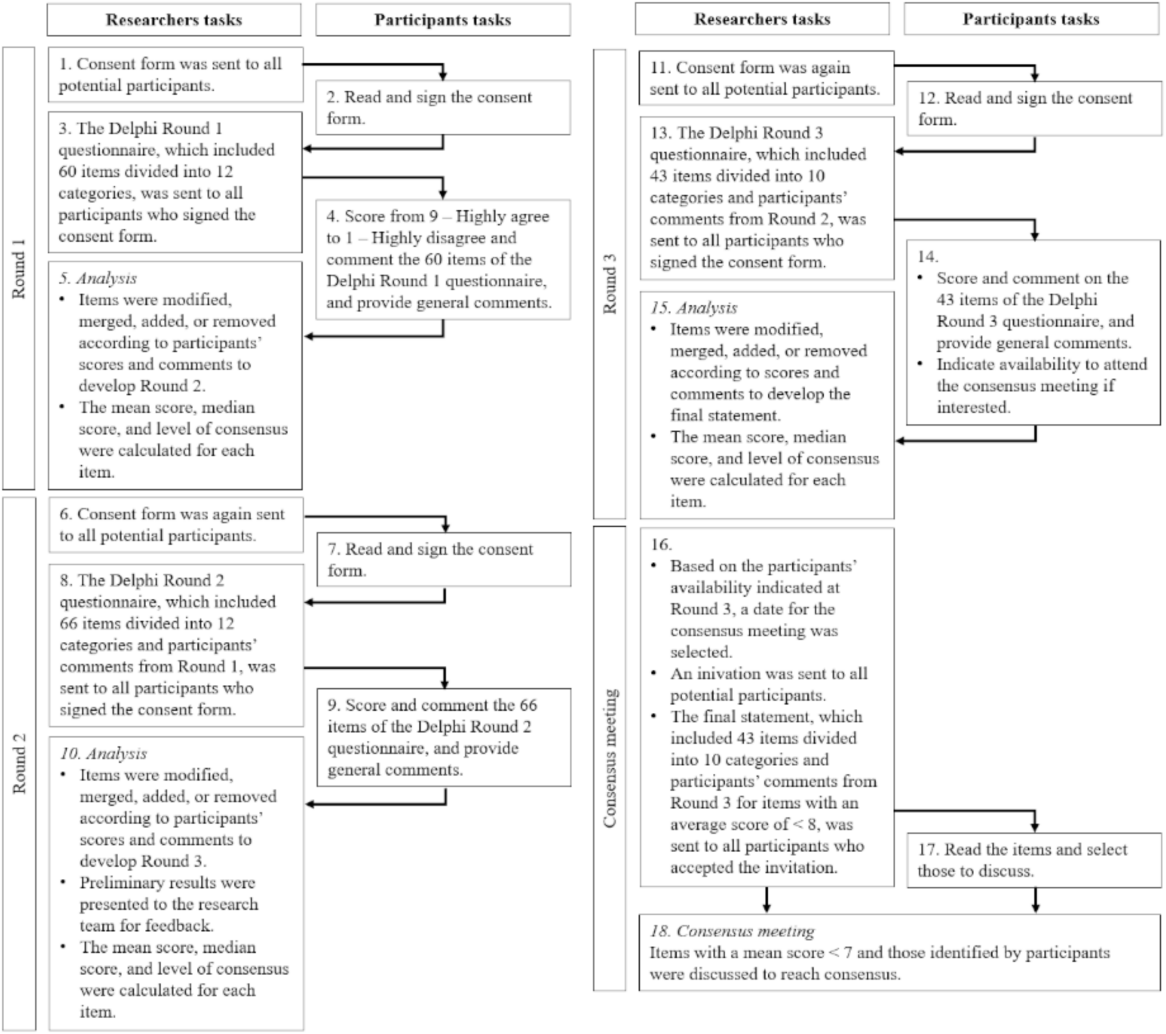
Delphi rounds and consensus meeting process.

Data were collected in three rounds between November 2024 and October 2025 (see **Figure 1**). For each round, an online information and consent form was distributed to potential panelists via a LimeSurvey (32) questionnaire. Once the form was signed, panelists promptly received the link to the anonymous Delphi questionnaire hosted on LimeSurvey. All materials were provided in English. To minimize attrition, a common challenge in Delphi studies (33), panelists had four weeks to respond to each questionnaire, and up to four reminders were sent to encourage completion. Experts with lived experience received financial compensation of 20 CAD, provided through an online gift card.

In Round 1 (completed in December 2024), panelists rated each item on a 9-point Likert scale, ranging from 1 (“highly disagree”) to 9 (“highly agree”), except for one item, which asked panelists to rank the values of forensic mental health services in order of priority.^1^ Panelists had the option to add comments and suggest additional items. Rounds 2 (completed in February 2025) and 3 (completed in October 2025) consisted respectively of 66 items across the 12 thematic areas and 43 items across 10 thematic areas. There was a longer interval between Rounds 2 and 3 than between earlier rounds. This delay was due to technical issues related to the survey platform at the institutional level, which were outside the control of the research team. During this period, preliminary findings were also reviewed with the Special Interest Group on Service Development and Delivery of the IAFMHS (June 2025). Items were edited based on feedback from the previous round, and panelists viewed a histogram representing how people voted in the previous round, as well as viewed comments from the previous round. They could see the initial item and the modified item. They were again invited to rate their agreement from 1 to 9.

#### Consensus meeting

A 90-minute consensus meeting was held in November 2025, and facilitated by the first two authors. Prior to the meeting, panelists were provided with a draft consensus statement, which they were asked to review in advance. The draft statement was color-coded to indicate the final level of agreement for each item: red indicated an average score below 7, orange represented scores between 7 and 7.9, and green denoted scores of 8 or above. Items that did not reach consensus or those marked in red (average score < 7) were prioritized for discussion during the meeting. At the beginning of the meeting, panelists were given 5 minutes to review the consensus statement and raise any items they wished to discuss further. The goal of the meeting was to address areas where consensus had not been reached and to refine the final consensus statement based on group feedback. Items were revised and workshopped by the whole group during the meeting, with all items reaching consensus.

### Analysis

In line with the Core Outcome Measures in Effectiveness Trials (COMET) guidelines, consensus was defined as ≥75% of panelists rating an item between 7 and 9 (34, 35). We also calculated mean scores for each item. The first two authors reviewed panelists’ responses to prepare subsequent rounds, including items that had already reached consensus. Items were reformulated, merged when there was repetition, and new ones were added upon suggestions.

Finally, the panelists’ responses were analysed using content analysis (36). We used a predefined analytic framework comprising five categories: (1) conceptual clarity or understanding, (2) scope and contextual applicability, (3) normative considerations, (4) operationalization or feasibility, and (5) “other” for non-analytic comments. *Conceptual clarity or understanding* refers to comments indicating confusion, ambiguity, or concerns about wording and definitions. *Scope and contextual applicability* refers to comments that highlight variations across legal frameworks, jurisdictions, and other service structures that could influence the relevance or applicability of an item. Normative considerations refers to comments about what constitutes appropriate, legitimate, or ethical practice in forensic mental health services. Finally, operationalization or feasibility includes comments focused on implementation and practical constraints, including procedures and resources. Each comment was assigned a single primary category based on the dominant reason articulated for ratings. This qualitative analysis was explicitly process-focused and subordinate to the quantitative Delphi results. We aimed to clarify how consensus emerged, the process to revise items, and why disagreement persisted across rounds, rather than generating standalone qualitative themes (36, 37).

### External validation

The pre-final statement was sent to the 69 members of the special interest group in Service Delivery and Development of the IAFMHS to obtain feedback and ensure the validity, applicability, and clarity of the standards. Three members provided written feedback. Their comments were reviewed and integrated where they contributed to clarifying wording, improving precision, or strengthening alignment with the existing consensus. One suggestion, which proposed the addition of a new item, was not incorporated, as it fell outside the scope of refinement at this stage of the consensus process.

## Results

### Round 1

From a quantative standpoint, the average agreement score for the first round was 8.15 (*SD* = 0.50), with scores ranging from 7.11 for the definition to 8.77 (*SD* = 0.03) for the restrictive measures theme (see **Table 2** for a mean score by theme). In Round 1, all but one item (59/60) reached consensus, defined as over 75% agreement. However, the item concerning the inclusion of peer support workers and the integration of a lived experience workforce as part of the care team at all stages of treatment did not. While many panelists felt that a lived experience workforce was essential, others raised concerns regarding feasibility, its importance (“*valuable but not essential*”), or the “*very limited*” evidence base. Others raised concerns about confidentiality: *“My reason for disagreement is around lived experience workers being an integral part of the MDT meaning they would have access to confidential information without a professional training”*.

**Table 2:** Summary of mean item score within theme.

Theme	Round 1 (k = 60)		Round 2 (k = 66)		Round 3 (k = 43)	
	Mean (SD)	Disagreements (N)	Mean (SD)	Disagreements (N)	Mean (SD)	Disagreements (N)
Definition	7.11	0	7	1	7.31	0
General statement	NA	NA	NA	NA	7.62	0
Model of care	7.76 (0.13)	0	7.54 (0.27)	1	7.89 (0.04)	0
Values	7.81	0	7.2	0	NA	NA
Pathways and processes	7.45 (0.23)	0	7.74 (0.51)	0	7.49 (0.64)	1
Programs, treatments, and activities	7.99 (0.41)	1	8.37 (0.38)	0	8.41 (0.46)	0
Physical health	8.50 (0.41)	0	7.91 (0.41)	1	8.46 (0.23)	0
Service users, peers, and loved ones	8.65 (0.09)	0	8.42 (0.14)	0	7.89 (0.12)	0
Evaluation and improvement	8.54 (0.28)	0	8.32 (0.22)	0	8.05 (0.22)	0
Collaboration between services	8.36 (0.33)	0	8.28 (0.44)	1	8.37 (0.36)	0
Safe environment	8.53 (0.07)	0	8.57 (0.20)	0	8.36 (0.45)	0
Restrictive measures	8.77 (0.03)	0	8.35 (0.23)	0	8.42 (0.35)	0
Other	8.28 (0.19)	0	8.13 (0.31)	0	8.21 (0.22)	0
**Total**	**8.15 (0.50)**	**1**	**7.99 (0.49)**	**4**	**8.04 (0.38)**	**1**

Scores ranged from 1 (“highly disagree”) to 9 (“highly agree”). Consistent with the Core Outcome Measures in Effectiveness Trials (COMET) guidelines, consensus was defined as ≥75% of panelists rating an item between 7 and 9 (34, 35). Bolded values represent overall mean items scores and standard deviation, as well as the total number of disagreements for each round.

From a qualitative standpoint, a total of 314 comments were analyzed in round 1. Approximately half (52%) focused on operationalization or feasibility, while 32% addressed normative considerations (see **Table 3**). The remainder of the feedback was categorized as contextual applicability (5%), conceptual clarity or lack of understanding (5%), or other (6%). Among the comments associated with ratings in disagreement with the item (those with a score <7), 51.2% were related to operationalization and feasibility issues, 22.0% to normative/value-based concerns, 17.1% to conceptual clarity issues, and 7.3% to contextual applicability.

**Table 3:** Type of comments at each round.

Type of comments	Round 1 n (%)	Round 2 n (%)	Round 3 n (%)
Normative considerations	101 (32%)	118 (33%)	58 (36%)
Operationalization and feasibility	162 (52%)	72 (20%)	16 (10%)
Scope and contextual applicability	17 (5%)	11 (3%)	2 (1%)
Conceptual clarity and understanding	15 (5%)	108 (30%)	49 (30%)
Other	19 (6%)	52 (14%)	36 (22%)
*Total*	314 (100%)	361 (100%)	161 (100%)

Panelists raised a range of concerns, which were used to further refine the items, regardless of the score associated with the comment or the mean rating of the item. From a normative perspective, panelists highlighted the challenges in balancing public safety with therapeutic goals, with some expressing concerns that an emphasis on recovery-oriented and person-centered approaches might interfere with broader safety or rehabilitation objectives. Additionally, panelists suggested including the importance of gender-responsive, culturally competent care, spiritually appropriate practices, greater incorporation of lived experience expertise, and a commitment to shared decision-making. For example: “*Definition needs to include: culturally safe, gender responsive, trauma informed alongside ‘safe and effective’, as fundamental components*” or “*I wonder if something about the family or loved ones should not be included in what should be high quality services*”.

In terms of operationalization and feasibility, several comments pointed to the limited evidence base for some of the proposed approaches (e.g., lived experience workforce), while others proposed that prioritizing evidence-based interventions could unintentionally favor short-term, easily measurable outcomes over more holistic care: “*The danger in promoting evidence-based practice in treatment programmes is that there is a focus on short-term, cheap, easily studied interventions rather than lengthy, relational, individualized, culturally relevant, spiritually appropriate, holistic development*”. Concerns were also raised about resource constraints and operational risks, particularly in relation to security and confidentiality.

Finally, comments on scope and contextual applicability mainly called for adjustments to account for varying legal frameworks, system organizations, and population heterogeneity, ensuring that the model can be tailored to fit different settings and cultural contexts.

In response to feedback from the first round, the items were revised to adopt increasingly clear, careful and context-sensitive wording. Given this was an international delphi project, revisions were especially attentive to cultural discrepancies and intended to accommodate variation across jurisdictions. We also expanded the explicit inclusion of value-based and person-centered principles identified by panelists as under-specified in the initial items. As a result, the second round of the Delphi included 66 items. Of the 60 initial items, 3 were removed, 45 were modified, 2 were merged to form 1 item, 10 were added, and 10 remained unchanged.

### Round 2

The average agreement score for the second round was 7.99 (*SD* = 0.49), with scores ranging from 7 for the definition to 8.57 (*SD* = 0.20) for safe environments. Of 66 items four did not reach consensus, in the following themes: definition, model of care, physical health, and collaboration between services.

In Round 2, the distribution of comments (*n* = 361) shifted substantially compared to Round 1. Normative considerations accounted for 33% of comments (*n* = 118), whereas those related to operationalization and feasibility represented 20% of feedback *(n* = 72), which was a substantial decrease compared to Round 1. Conceptual clarity and understanding emerged as a prominent category in Round 2, accounting for 30% of comments (*n* = 108). Finally, comments related to scope and contextual applicability were infrequent (3%, *n* = 11) and comments categorized as “Other” represented 14% of feedback (*n* = 52) and were overwhelmingly associated with high agreement (93% rated 7–9). Among the comments associated with ratings in disagreement with the item (those with a score <7), 42% were related to conceptual clarity, 32% to normative concerns, 19% to operationalization, and 4% to contextual applicability.

Comments related to conceptual clarity included concerns that the items had become too verbose: “*There are a lot of positive features listed here, but it reads like a laundry list rather than a coherent vision for services*.” Panelists also questioned whether some items described existing practices or aspirational goals. No substantively new normative concerns were introduced in Round 2, although tensions were made stronger in response to panelists' comments in Round 1, which were provided along with revised items: “*We need to be aware that peer experts are not inherently fragile, while clinicians are not inherently robust. A fear that peers will act unprofessionally or become distressed is not an adequate argument against their contribution, and comments of that nature illustrate why a peer workforce is desperately needed (not least to educate clinicians).”* There was also some disagreement about the space that spirituality should occupy: “*I come from a secular country where religion is a private matter.”*

Feasibility concerns increasingly focused on the implementation consequences of expanded scope and wording, with panelists emphasizing the need for prioritization and adaptation to local constraints. For instance, one participant noted: *“I think the second version is very humane, but it sounds like a lot of people [would] need to be informed, trained, coordinated, etc. If we overcomplicate the system, it becomes dysfunctional.”* In contrast, other panelists explicitly cautioned that such feasibility-driven qualifications allow core commitments to be deprioritized: *“It’s disheartening to see how easily comments can fall into the trap of agreeing, with heavy qualifications [ … ]. It is very easy for guidelines to be read as recommendations and ignored. Recovery, trauma-informed care, family involvement, etc., are too often afterthoughts after security, and are never actually implemented in practice.”*

In light of this type of feedback, several changes were made to items and also to the overall structure of the set of criteria. Specifically, we created distinct items to distinguish between a definition of existing forensic mental health services and aspirational principles (**Table 4**). Principles that had been repeated across multiple items (e.g., recovery-oriented, person-centered) were consolidated into a single set of general principles. In addition, qualifying language that risked rendering principles optional (“if appropriate”) was reduced. As a result, the third round of the Delphi included 43 items 2 were added, 18 were removed, 29 were modified, 10 were merged to form 3 items, and 9 remained unchanged.

**Table 4:** Two examples of the evolution of statements from round 1 to the final version.

Item	Round 1	Round 2	Round 3	Final version
Definition	Forensic mental health services provide person-centred, public protecting, safe and effective assessment, care and treatment for persons with mental health disorders who pose a risk of harm to others; and have come to the attention of the justice system (or their behaviour poses a risk of such contact); in conditions of therapeutic safety and security in hospital, the community or custody settings.	Forensic mental health services aim to provide evaluation, care, and treatment for persons with mental health problems who pose a risk of harm to others; and have come to the attention of the justice system (or their behaviour poses a risk of such contact); in conditions of therapeutic safety and security in hospitals, correctional settings, communities or custody settings. These services should be person-centred, recovery-oriented, culturally safe, gender responsive, trauma-informed, community reintegration oriented, inclusive of families and loved-ones, public protecting, and grounded in safe and effective assessment.	Forensic mental health services provide evaluation and treatment for persons with mental health problems who pose a risk of harm to others; and have come to the attention of the justice system (or their behaviour poses a risk of such contact); in community and institutional settings across health, social, and correctional sectors.	Idem
Lived experience workforce	Peer support workers or a lived experience workforce should be prioritized, involved in all stages of development services and environment, and be an integral part of a multidisciplinary team. Peer support programs should be available to service users within forensic mental health services, leveraging the lived experience of peers to foster hope, empowerment, and recovery.	A lived experience workforce, including advisors and peer support workers should be involved at all stages of service and environment development and serve as integral members of the multidisciplinary team. Their contributions, grounded in lived experience, should be leveraged to foster hope, empowerment, and recovery among service users. Clear guidelines should delineate their responsibilities, ensuring they collaborate with the care team while maintaining their distinct roles and are not used to impose coercion on service users.	A lived experience workforce, including advisors and peer support workers, should be involved at all stages of service and environment development and serve as integral members of the interdisciplinary team throughout the entire care process, including after discharge. Their contributions, grounded in lived experience, should be leveraged to foster hope and empowerment among service users.	A lived experience workforce, including advisors and peer support workers, should be involved in all aspects of care, as well as service development and delivery. Their contributions, grounded in lived experience, should be leveraged to foster hope and empowerment among service users, including after discharge.

### Round 3

The average agreement score for the third and final round was 8.04 (*SD* = 0.38), with scores ranging from 7.31 for the definition to 8.46 (*SD* = 0.23) for physical health. One item out of the 43 items did not reach consensus. This sole item related to the prioritization of the wisdom of cultural leaders in decision-making.

There were a total of 161 comments, of which 93% were associated with a score greater or equal to 7. Similar to the other rounds, normative comments represented approximately a third (36%, n = 58) of all comments; conceptual clarity and understanding represented another 30% (n = 49); operationalization and feasibility constituted 10% (n = 16); and the remainder fell under the "Other" (22%, n = 36) and scope and contextual applicability (1%, n = 2) categories. Among the comments associated with ratings in disagreement with the item (those with a score <7), 42% were related to conceptual clarity, 33% to normative concerns, 8% to operationalization, 4% to contextual applicability, and 17% for other reasons (see **Table 3**).

Overall, comments in Round 3 were largely supportive of the revised items or focused on further refinement, rather than introducing new ideas or raising additional concerns. One area that continued to generate debate, however, concerned the inclusion of cultural leaders’ knowledge and wisdom, specifically in the context of risk assessment. Several panelists supported explicitly valuing Indigenous and cultural practices: “*The wisdom of cultural leaders should be given special weight, as structured professional judgement tools that fully understand Indigenous populations, spiritual factors, etc., do not currently exist.”* However, others cautioned against this approach: “*You can’t have cultural leaders over-calling a risk assessment tool. They can contribute but their view cannot be prioritized over a validated scientific approach.”* These exchanges highlight ongoing disagreement regarding what constitutes evidence-based practice and how culture should be integrated into forensic mental health models, with comments ranging from concerns about the potential reinforcement of harmful cultural norms (“*what if the patient’s culture is homophobic or does not respect women?*”) to critiques of what were perceived as culturally insensitive positions expressed by other panelists.

### Consensus meeting

Seven panelists participated in the consensus meetings. Non-attendance was primarily due to scheduling constraints across multiple international time zones. In total, five items were discussed: the prioritization of cultural leaders in structured professional judgment; the management of transfers between levels of care; the role of a lived experience workforce; the collaboration with general mental health; and restorative justice. The first two were discussed based on the scores at Round 3, while the last three were discussed at the request of panelists. Four of these items were revised and subsequently reached consensus. Following discussion, panelists agreed not to revise the restorative justice item and retain the Round 3 version. Revisions focused on reducing prescriptive formulations and increasing flexibility. This included removing contested formulations (e.g., “prioritize”) and replacing specific mechanisms with broader processes (e.g., shifting from admission and review committees to standardized, transparent transition processes), as well as softening rigid role definitions (e.g., reframing lived experience roles as involvement in care and service development rather than as integral members of all interdisciplinary teams).

In addition, the general principles were refined, with two principles added to the general statement, to explicitly address the needs of service users with cognitive impairments, and the right to culturally safe care for Indigenous service users and ethnic minorities, with the wisdom of cultural leaders being included in all decision-making. The principle related to family involvement was also revised to reflect that support may not come from family members and should instead refer to a chosen support network.

### Consensus statement

The Consensus Statement is organized into three main components. First, it includes an overarching definition of what forensic mental health services *are*:

“Forensic mental health services provide evaluation and treatment for persons with mental health problems who pose a risk of harm to others; and have come to the attention of the justice system (or their behaviour poses a risk of such contact); in community and institutional settings across health, social, and correctional sectors.”

This definition is followed by 12 guiding principles indicating what forensic mental health services *should be* (see **Table 5**). These include commitments to (1) a rights-based approach; (2) comprehensive safety, including relational security; (3) recovery orientation; (4) trauma-responsive care; (5) person- and collectivity-centered care; (6) inclusion of a chosen support network; (7) stigma reduction; (8) culturally safe care for Indigenous people and ethnic minorities; (9) gender-responsive practice; (10) responsiveness to cognitive impairments; (11) evidence-based care; and (12) physical health and wellness.

**Table 5:** Consensus statement: 12 guiding principles.

Principle	Description
Rights-based approach	Forensic mental health care must uphold and actively support the rights of service users.
Comprehensive safety	In addition to physical and procedural security, relational security including both quantitative aspects (e.g., skills mix, training) and qualitative aspects (e.g., maintaining therapeutic relationships, safe boundaries, and trust), should be provided to ensure comprehensive and effective service user care.
Recovery orientation	Forensic mental health services should be recovery-oriented by fostering social connections, nurturing hope and optimism for the future, supporting the rebuilding of a positive identity, facilitating meaningful engagement in life, and empowering service users to take control of their recovery journey.
Trauma-responsive care	Services must be grounded in an understanding of how trauma affects individuals, recognize signs of trauma, implement systems that prevent retraumatization, and provide comprehensive training on trauma-informed practices.
Person- and collectivity-centered care	Service users should be actively involved in all levels of care ensuring their preferences and goals are prioritized while acknowledging power dynamics. Services must also be tailored to collective needs, with explicit attention to equity, diversity, inclusion, and citizenship.
Inclusion of a chosen support network	Forensic mental health services should facilitate the active involvement of a chosen support network, including family and friends, in the treatment and care of service users, with the service user’s consent. This includes participation in treatment planning, progress reviews, and discharge planning to ensure continuity of care and support. Clear guidelines should be established to frame and support this practice.
Stigma reduction	Forensic mental health services should actively address structural and interpersonal stigma through strategies that challenge negative stereotypes and promote inclusive, respectful environments. These services should also equip service users with tools to manage stigma, empowering them through education, peer support, and advocacy initiatives.
Culturally safe care for Indigenous people and ethnic minorities	Service users should have access to culturally appropriate care that respects their cultural needs and reflects their identity and values by incorporating cultural practices and frameworks into their treatment to enhance their well-being and recovery. The wisdom of cultural experts should be included in all decision-making, as existing practices and frameworks may not fully account for cultural needs, including those of Indigenous communities.
Gender-responsive practice	Forensic care should be developed to address the specific needs of women and non-binary service users. They should be involved in all stages of developing a safe and fulfilling environment that meets these needs.
Responsiveness to cognitive impairments	Services should assess and respond to the cognitive needs of service users. It should be acknowledged that, in some jurisdictions, forensic mental health service users may include those with intellectual disabilities, who have distinct needs.
Evidence-based care	Services should prioritize effective evidence-based care. In the absence of established evidence, care should be principles-based and aimed at generating new evidence.
Physical health and wellness	Services should also provide health promotion and disease prevention programs, including education on nutrition, lifestyle, family health, and sexual and reproductive health. Diverse and easily accessible physical activity programs should also be integrated into treatment plans to promote physical fitness, reduce stress, improve overall mental health, and lower the risk of chronic diseases. Service users should be encouraged to make healthy choices.

Following these guiding principles, 43 core components of forensic mental health systems are grouped across 10 thematic domains (see **Table 6**): (1) Model of care; (2) Pathways and processes; (3) Programs, treatments, and activities; (4) Physical health; (5) Service user, peer, and carers involvement; (6) Evaluation and improvement; (7) Integration and collaboration between services; (8) Safe environment; (9) Restrictive measures; and (10) Other.

**Table 6:** Consensus statement: 43 core components.

Model of care
1. Forensic mental health services should have a documented model of care with a clear rationale and include a logic model that provides guidelines for delivering concrete care to achieve the objectives. This model of care should be regularly updated in close collaboration with all stakeholders. 2. Models of care in forensic mental health should be rigorous, methodical, capable of objective evaluation, humanistic, holistic, and evolving according to new practices. 3. Forensic mental health services should provide population-based levels of service (assessment and treatment) that are sustainable with an emphasis on long-term planning. 4. Balanced care systems should be developed to meet the diverse needs of forensic mental health service users, ensuring a mix of secure and community-based services, and aligning service intensity with the severity of service users’ mental health and substance use problems, as well as their risk to themselves and others.

Pathway and Processes
5. Clear and efficient pathways should be established to facilitate transitions between forensic mental health, criminal justice, general mental health, and other relevant services (e.g., family practitioners, social services, first responders). These pathways should include systematic prison screening, court liaison, diversion services, clear allocation, and waiting-list management. Seamless continuity of care should be supported through written guidelines, interdisciplinary meetings, shared protocols, and coordinated care plans. 6. Structured professional judgment frameworks should be used in forensic mental health services and should be validated for specific populations. 7. Services should be conducting effective and objective assessments for the courts and other parties requesting a forensic evaluation. 8. Forensic care frameworks should be supported by regular interdisciplinary clinical, cultural, and lived experience audits to ensure the sustainability, quality consistency, and continuous improvement across the service. 9. Standardized processes based on clinical progress should be used to guide transitions, ensuring informed and transparent transitions. 10. Detailed processes for managing service user discharge should be in place in forensic mental health services. These processes should include collaborative discharge planning as well as follow-up meetings and systematic procedures to ensure consistency, compliance, and continuity of care. 11. Services must actively manage length of stay, ensure continuity across the care pathway, and maintain the sustainability of service capacity for the forensic population. 12. Specialist management and governance structures should be established in forensic mental health services, with representation from all stakeholders. These structures should be clearly documented and subject to external review. They should include clear lines of reporting and responsibility, processes for regular monitoring and benchmarking of key decisions (admission, transfer, treatment, discharge) as well as quality of care, and responsiveness to complaints according to predetermined criteria, compliance with legal and policy requirements, and the maintenance of inter-agency relationships.

Programs, Treatments, and Activities
13. Evidence-based multimodal treatment programs should be available to address the complex needs of forensic mental health service users, including violence prevention, mental health, substance misuse, and offending behaviors. 14. A lived experience workforce, including advisors and peer support workers, should be involved in all aspects of care, as well as service development and delivery. Their contributions, grounded in lived experience, should be leveraged to foster hope and empowerment among service users, including after discharge. 15. Interdisciplinary teams, including psychiatrists, psychologists, nurses, social workers, peer support workers, cultural advisors, and other allied health professionals, should be employed to provide comprehensive care in forensic mental health services, ensuring that each discipline contributes to the individual care and treatment plan. 16. Forensic mental health services should offer meaningful activities for service users everyday of the week to support them in adopting a (more) prosocial identity. Meaningful activities are defined as activities freely chosen that align with a person’s interests, preferences, abilities, goals, and identity. 17. Service users should be provided with the opportunity to attend school, engage in work, or pursue vocational training. 18. Treatment for substance use disorder must be available and easily accessible. 19. Forensic services should aim to support service users to fulfill primary needs (e.g. creativity, inner peace, relatedness, agency, spirituality) according to their preferences. 20. When all parties are willing, forensic mental health services should explore restorative justice as a pathway to foster accountability and recovery for those who caused harm while promoting healing for those affected. Further research is needed to strengthen the evidence base for this practice.

Physical Health
21. Nutrition and dietitian services should be accessible to service users in forensic mental health settings, providing dietary assessments, tailored nutrition plans, and education on healthy eating habits and lifestyles. Additionally, service users should receive support to transition to self-catering. 22. Service users should be allowed to have an intimate relationship if they desire to. They should receive support through education, access to specialized professionals, counselling, and institutional policies that respect their rights.

Service Users, Peers, and Carers Involvement
23. Forensic mental health services should offer support and education to carers, helping them understand the service user’s condition and treatment while equipping them with the skills to offer effective support, all done with full transparency with the service user. 24. Service users should have access to their medical files in a clear, timely, and transparent manner and be encouraged to consult them. Medical files should include the service user’s point of view, and be presented in a comprehensible and accessible way.

Evaluation and Improvement
25. Forensic mental health services should regularly evaluate their models of care, services, and programs through external audits, feedback mechanisms, and performance comparisons against national and international standards of quality and excellence. These evaluations should rely on standards developed in collaboration with other services, as well as established performance indicators. The efficiency and effectiveness of care programs should be assessed for potential adverse effects and near-miss scenarios, with adjustments made based on clinical evaluation findings. 26. Resilience should be a key focus in forensic mental health services (i.e. resilience is the capacity of health systems to proactively foresee, absorb, recover from, and adapt to shocks or changes), involving proactive planning, adaptive capacity building, and strategic resource allocation to effectively respond to new demands, crises, and challenges while maintaining service continuity and quality. 27. Comprehensive and multidimensional outcome measurement should be implemented in forensic mental health services, evaluating humanitarian, psychosocial, health, and public safety outcomes, and taking into account the perspectives of multiple interested groups.

Integration and Collaboration Between Services
28. Clear processes for inter-agency relationships and boundaries should be established in forensic mental health services, including formal agreements, defined roles and responsibilities, and communication protocols to facilitate effective collaboration and prevent service gaps. 29. Forensic mental health services should collaborate with general adult mental health services, creating shared care pathways and integrated service models to provide continuous and holistic care for service users across different service settings. 30. Forensic mental health services should work closely with housing and social service agencies to support service user reintegration, ensuring access to stable housing, social support, and vocational opportunities as part of comprehensive discharge planning. 31. Continuity of care should be maintained throughout the forensic mental health services pathway with consistent planning of care and treatment that aims to maximize service users’ personal agency, ensure successful community supervision, and reduce risk to self and others.

Safe Environment
32. High staff-to-service user ratios, which take into account skills, gender, and cultural diversity, should be maintained in forensic mental health facilities. This ensures sufficient staffing levels to provide comprehensive care and manage challenging behaviors effectively. This includes maintaining ratios that allow for consistent and meaningful service user engagement, ensuring both relational safety and quality of care. 33. The physical environment in forensic mental health facilities should be designed to promote security while also offering a homely (e.g.,, single and personalised rooms) and stimulating atmosphere by incorporating features such as secure perimeters, ligature-free environments, clear sightlines, natural daylight, and access to outdoor spaces. The environment should be robust, escape-proof, and aesthetically pleasing to support service user well-being and safety. 34. Hospital security procedures should include systematic and continuous risk assessment and management by implementing policies and practices for controlling risk, regular monitoring of service user movements and communication, staff training, professional governance, dynamic risk assessments using validated tools, and risk management strategies. 35. Systems for incident reporting and organizational learning should be in place in forensic mental health services, including standardized procedures for documenting incidents, analyzing shortcomings, and implementing corrective actions to prevent recurrence and enhance service quality. 36. Comprehensive evidence-based interventions should be implemented to prevent suicide in forensic mental health services. Staff should receive regular training to effectively identify and address suicide risks. Safe, positive environments that promote well-being should be fostered. Collaborative care plans should enhance protective factors and encourage positive risk taking.

Restrictive Measures
37. Restrictive measures such as restraint and seclusion should only be used as a last resort in forensic mental health services, authorized only when service users pose an imminent and serious risk of harm to themselves or others. They should last only as long as the active risk lasts and should be followed by appropriate and voluntary debriefing and support. 38. Restrictive measures must be carefully regulated and implemented in compliance with strict legal and ethical guidelines, such as human rights legislation. Advance care planning developed in collaboration with the service user should be implemented. Additionally, the role of peer support and lived experience should be considered when evaluating and applying restrictive measures, ensuring that the approach remains informed, and compassionate. 39. Service providers should ensure that systems are in place to monitor the side effects, vital signs, hydration levels, and consciousness of service users under restrictive measures. 40. Healthcare practitioners should be trained in the application of de-escalation techniques and effective communication. 41. Forensic mental health services should provide therapeutic spaces designed to help service users stabilize before and/or after an incident or intervention. They should be accessible to service users to help them find relief when early warning signs emerge. These spaces should offer close monitoring, and provide support from trained cultural and peer workers. They should also facilitate a calm transition back to the ward, guided by clear policies and individualized care plans.

Other
42. Spirituality should be recognized as an important aspect of care in forensic mental health services. They should support service users’ spiritual needs in a respectful, non-coercive manner and ensure access to spiritual support. 43. Forensic mental health services must ensure that service users have access to effective advocacy mechanisms, including a responsive complaints system that enables them to raise concerns clearly, confidentially, and accessibly, as well as timely access to legal representatives. These mechanisms should ensure that service users’ voices and legal rights are respected and upheld throughout their care and treatment. All concerns should be addressed promptly, fairly, and transparently, through processes that remain independent of the care team.

While intended to support international dialogue, these Standards of Care may be most directly applicable to jurisdictions with established forensic mental health systems; adaptation may be required in contexts where such structures are emerging.

## Discussion

The present Delphi consensus building process highlights the extent to which forensic mental health remains a field shaped by persistent tensions. Although high levels of quantitative agreement were reached across all rounds and the vast majority of statements, the accompanying qualitative comments revealed areas of persistent disagreement requiring ongoing consideration and negotiation. This underscores the importance of qualitative engagement in contested fields, where consensus may overshadow underlying tensions (38–40). In the present study, the Delphi process made visible a recurring gap between the values forensic mental health services explicitly endorse and the values that implicitly govern practice (41, 42). Principles related to recovery orientation, cultural safety, lived experience, and family and community involvement were widely endorsed by panelists; yet, their implementation was constrained by implicit values, such as risk minimization, public safety, accountability, and professionally defined notions of service users’ best interests.

These tensions between explicit and implicit values cannot be fully understood outside the historical legacies that continue to structure forensic mental health. First, forensic mental health has developed at the intersection of psychiatry and criminal law (3), and this medico-legal foundation shapes contemporary practice (43). The primacy of public safety and risk assessment remains central to how services are organized, which limits the integration of recovery-oriented and rights-based principles into practice (1). This legacy was evident in panelists’ concerns about decision-making authority in matters involving risk, as well as ambivalence regarding the role of peer support workers. Second, the field is grounded in Western positivist epistemologies (44), which privilege standardized and measurable forms of evidence (42). Within this framework, structured risk assessment tools were ascribed high legitimacy, while other forms of knowledge (cultural, spiritual, experiential) were often viewed as less rigorous. Tensions between “evidence-based” and more values-based or experience-informed approaches (42) therefore reflect deeper questions about what counts as valid knowledge in forensic mental health (17). Finally, these epistemic tensions intersect with broader Western and colonial legacies around autonomy, confidentiality, and privacy. European notions of autonomy and privacy influenced how culture and spirituality were viewed, often positioning them as personal or “private” matters rather than a foundational element of care and healing. This may explain why some panelists expressed discomfort with integrating cultural or spiritual practices, even within secure settings where autonomy and privacy are already substantially constrained.

While it is possible that the international composition of the panel added layers of complexity that contributed to dissensions, the marked decline in comments related to scope and contextual applicability across rounds, compared to the persistence of normative considerations, instead suggests that enduring value-based tensions, rather than contextual heterogeneity, underpinned persistent disagreement. In this context, consensus was possible largely because value-based commitments were articulated as guiding principles rather than as fully operationalized requirements. Panelists were able to agree on the foundational aims and core values of forensic mental health services even when they disagreed on how they should be enacted in practice. This pattern reflects the coexistence of explicit and implicit values within forensic mental health systems, and the dominance of implicit values in the context of competing priorities (41). This raises the question of whether the principles should be treated as optional aspirations or as integral standards of care. In the present framework, these principles are positioned as normative expectations from which deviation should remain exceptional, under clearly justified circumstances, and transparent. Yet, in many systems, these principles remain symbolic, expressed in mission statements without being subject to systematic evaluation or accountability mechanisms (45). For example, stigma reduction is frequently cited as a core commitment, but is rarely assessed through routine indicators, audits, or performance frameworks (46). This gap highlights a broader challenge at the intersection of values, implementation, and governance in forensic mental health.

### Implications

At the policy and practice level, this international consensus statement provides a shared reference point for clinicians, policymakers, system leaders, and decision-makers across jurisdictions. This is particularly important in forensic mental health, where services span health, social services, justice, and correctional sectors, and operate within heterogeneous legal and regulatory frameworks (1). By articulating a common definition, guiding principles, and core service components, the framework offers a shared language to align expectations across sectors and to support policy development, legislation, and accountability mechanisms. The consensus statement also has implications for how forensic mental health services are evaluated and held accountable. By explicitly articulating commitments related to recovery, rights, cultural safety, and lived experience, it broadens the scope of what systems might reasonably be assessed on. While public safety outcomes such as incidents and recidivism remain essential, they do not capture the full mandate of forensic mental health services. Just as oncology services are no longer evaluated solely on cancer-related mortality but also on indicators such as progression-free survival, treatment response, timeliness of care, user-reported outcomes, and equity of access (47), forensic mental health services should not be assessed only on recidivism or adverse events. Meaningful evaluation should also consider continuity of care, quality of life, recovery trajectories, respect for rights, cultural safety, and service user experience (48). This framework thus has direct relevance for monitoring, accreditation, and audit processes.

Finally, the consensus statement also has implications for research. Much of the existing literature on forensic mental health models of care remains descriptive (e.g., 14, 23), without evaluating their implementation or effects. This consensus statement provides a framework to develop indicators and to support comparative evaluative research across jurisdictions.

### Limitations

This study has several limitations that should be considered when interpreting the findings and the resulting consensus statement. First, although the panel was international, panelists were predominantly based in Western, high-income countries. Forensic mental health services are historically rooted in European and Anglo-Saxon legal and psychiatric traditions, which continue to dominate professional institutions and knowledge production (17). The consensus articulated here reflects these dominant traditions rather than a global perspective. Second, all survey materials were developed and administered in English, which may have limited participation from non-English-speaking experts, especially amongst people with lived experience. Third, participants were recruited through established professional and service user networks, including the International Association of Forensic Mental Health Services and formal service user advisory committees. While this approach facilitated the inclusion of clinicians, researchers, managers, and individuals with lived experience who are actively engaged in service development, it may also have shaped the range of perspectives represented. Individuals connected to formal networks may be more involved in policy and system-level dialogue than those operating outside established structures. As a result, the panel may not fully capture perspectives from practitioners or people with lived experience who are not affiliated with professional or advisory bodies. Fourth, the number of panelists in this study was relatively modest (n = 23). While there are no agreed-upon standards for sample size in Delphi studies, some authors suggest a minimum of about 10–18 panelists (49), with around 30 carefully selected panelists considered ideal (50, 51). Finally, there was substantial attrition from recruitment to the final round. This attrition may have contributed to an inflated sense of consensus if panelists holding minority viewpoints were more likely to discontinue participation. Similarly, the reduced attendance to the consensus meeting may have limited perspectives represented during discussion. Although attrition is common in Delphi studies (33), particularly those involving time-intensive rounds, technical issues and platform-related challenges may have further contributed to participant fatigue and withdrawal.

## Conclusion

This Delphi consensus study offers an internationally informed framework to clarify what forensic mental health services are, what they should strive to achieve, and how they might be organized and evaluated. While the resulting consensus reflects dominant Western forensic traditions and the historical legacies that continue to shape the field, it also makes visible the persistent tensions between explicit commitments to recovery, rights, cultural safety, and lived experience, and the implicit priorities that structure everyday practice. As such, this statement should be understood as a starting point to support ongoing debate, adaptation, and evaluation across diverse forensic mental health contexts. It also sheds light on the challenges that must be addressed for forensic mental health services to evolve toward more transparent, equitable, safe, and recovery-oriented systems.

## Data Availability

The raw data supporting the conclusions of this article will be made available by the authors, without undue reservation.

## Footnotes

1 This item was aggregated with others in Round 3, and responses were also rated from 1 (“highly disagree”) to 9 (“highly agree”).
